# Intravenous leiomyomatosis is inclined to a solid entity different from uterine leiomyoma based on RNA‐seq analysis with RT‐qPCR validation

**DOI:** 10.1002/cam4.3098

**Published:** 2020-05-05

**Authors:** Wenze Wang, Yanfeng Wang, Fei Chen, Ming Zhang, Rujing Jia, Xingrong Liu, Chaoji Zhang, Jiang Shao, Ninghai Cheng, Guotao Ma, Zhaohui Zhu, Qi Miao, Zhiyong Liang

**Affiliations:** ^1^ Department of Pathology Peking Union Medical College Hospital Peking Union Medical College Chinese Academy of Medical Science Beijing China; ^2^ Department of Pathology Heilongjiang Province Land Reclamation Headquarter General Hospital Harbin China; ^3^ Department of Gynecology Peking Union Medical College Hospital Peking Union Medical College Chinese Academy of Medical Science Beijing China; ^4^ Department of Pathology Haidian Maternal & Children Health Hospital Beijing China; ^5^ Accreditation Dept Five (Proficiency Testing Dept.) China National Accreditation Service for Conformity Assessment (CNAS) Beijing China; ^6^ Department of Cardiac Surgery Peking Union Medical College Hospital Peking Union Medical College Chinese Academy of Medical Science Beijing China; ^7^ Department of Vascular Surgery Peking Union Medical College Hospital Peking Union Medical College Chinese Academy of Medical Science Beijing China; ^8^ Department of Nuclear Medicine Peking Union Medical College Hospital Peking Union Medical College Chinese Academy of Medical Science Beijing China; ^9^ Molecular Pathology Research Center Department of Pathology Peking Union Medical College Hospital Chinese Academy of Medical Sciences and Peking Union Medical College Beijing China

**Keywords:** angiogenesis, differentially expressed genes, high‐throughput whole transcriptome resequencing, intravenous leiomyomatosis

## Abstract

**Introduction:**

Intravenous leiomyomatosis (IVL) is currently regarded as a special variant of the common uterine leiomyoma (LM). Though IVL shows a similar histological morphology to LM, IVL is characterized by unique intravenous growth patterns and low‐grade malignant potential, which are quite different from LM. There are currently few studies underlying the molecular alterations of IVL, though this information is important for understanding the pathogenesis of the disease, and for identifying potential biomarkers.

**Method:**

We carried out a high‐throughput whole transcriptome sequencing of tumor and normal tissue samples from five IVL patients and five LM patients and compared the differentially expressed genes (DEGs) between IVL and leiomyoma. We performed multiple different enrichment and target analyses, and the expression of selected DEGs was validated using RT‐qPCR in formalin‐fixed samples.

**Results:**

Our study identified substantial different genes and pathways between IVL and LM, and functional enrichment analyses found several important pathways, such as angiogenesis and antiapoptosis pathways, as well as important related genes, including *SH2D2A, VASH2, ADAM8, GATA2, TNF,* and the lncRNA *GATA6‐AS1*, as being significantly different between IVL and LM (*P* = .0024, *P* = .0195, *P* = .0212, *P* = .0435, *P* = .0401, and *P* = .0246, respectively). *CXCL8, LIF, CDKN2A, BCL2A1, COL2A1, IGF1,* and *HMGA2* were also differently expressed between IVL and LM groups, but showed no statistical difference (*P* = .2409, *P* = .1773, *P* = .0596, *P* = .2737, *P* = .1553, *P* = .1045, and *P* = .1847, respectively) due to the large differences among individuals. Furthermore, RT‐qPCR results for five selected DEGs in IVL tissues and adjacent nontumor tissues were mainly consistent with our sequencing results.

**Conclusion:**

Our results indicated that IVL may be a solid entity that is unique and different from LM, proving consistent with previous studies. Furthermore, we identified DEGs, particularly within angiogenesis and antiapoptosis pathway‐related genes that may play crucial roles in the development and pathogenesis of IVL and may be potential specific biomarkers.

## Graphical abstract

A transcriptome sequencing on tumor and normal samples from five IVL patients and five LM patients was performed. Angiogenesis and antiapoptosis pathways, and some important genes, and lncRNA are vastly different between IVL and LM, with RT‐qPCR validation. The result suggests that IVL is a solid entity different from LM in the development and pathogenesis.

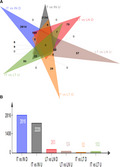

## 1 INTRODUCTION

**FIGURE 1:**
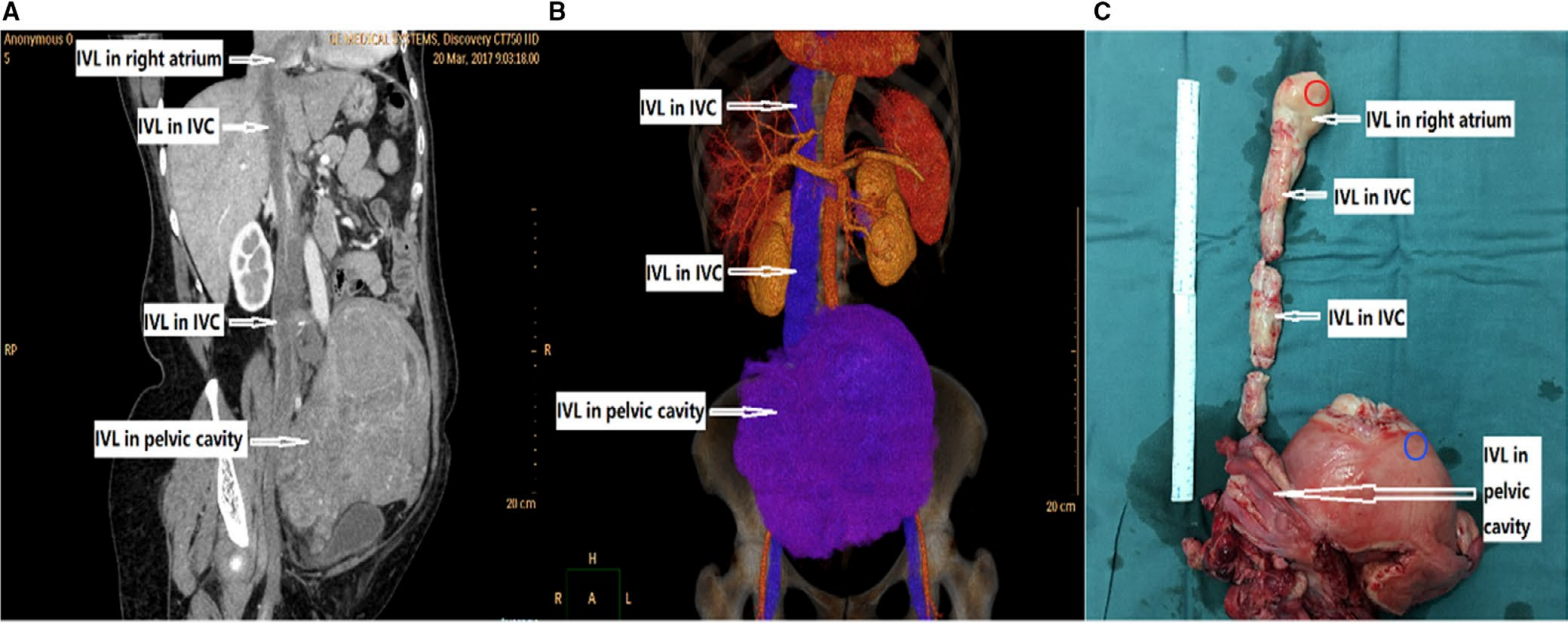
IVL clinical image. (A) Curved planar reformation (CPR) CT shows IVL in inferior vena cava (IVC) and pelvic cavity. (B) Volume rendering (VR) three‐dimensional reconstruction CT shows the 3D form of IVL before operation. (C) Specimen of IVL after operation. The red and blue circles indicate the location from where tumor and tumor‐adjacent normal tissue were collected, respectively

Intravenous leiomyomatosis (IVL) usually presents as a histologically benign smooth muscle cell tumor, growing from the uterus and invading into the extrauterine venous system, and is found to mainly occur in reproductive‐age women.^1^ Although histologically benign, IVL can grow into the iliac vein or ovarian vein, and sometimes extend to the inferior vena cava to reach the right heart chambers (Figure 1). Rarely, IVL can even extend into the pulmonary artery. In all of these cases, IVL can cause severe circulatory disturbances and even syncope or sudden death.^2^ IVL has no specific clinical symptoms or pathological imaging characteristics, so diagnosis prior to surgery can be very difficult. Furthermore, when IVL is confined to the uterus, it can be difficult for pathologists to differentiate it from LM. As such, IVL is often underestimated due to ease of misdiagnosis and the lack of specific identifying biomarkers.^3^

IVL has a unique and meandering pattern of growth within the blood vessels.^1^ As previous studies have suggested, the tumor surface is covered with a complete layer of vascular endothelial cells, and the microvascular density of the tumor is also higher than that of LM or normal uterine smooth muscle tissue.^3^, ^4^ IVL can even form a spongy network of vessels internally. This special growth pattern suggests that IVL has a strong induction of vascular endothelial cell proliferation and neovascularization. Given that IVL tends to keep growing within the vessel it is localized in, the mitosis and Ki‐67 indexes of IVL are usually low, indicating that IVL tumor cells may have high expression of antiapoptosis factors. As of yet, little is known about the underlying molecular pathological mechanism of this tumor.

LM is the most common tumor of the female genital tract, which presents nonrandom tumor‐specific cytogenetic abnormalities in about 25%‐40% of patients.^5^, ^6^, ^7^ In addition to the “usual” type of LM, there are also variants of LM, including, as of now, IVL. According to the WHO classification, LM is specified as ICD‐O code 0 (benign), while IVL (a variant of LM) is specified as ICD‐O code 1 (uncertain malignant potential). Another category of tumor, smooth muscle tumor of uncertain malignant potential, also is specified as ICD‐O code 1.^8^ This adds to the confusion of the diagnosis and treatment of IVL.

Although IVL is quite different from LM in clinical and pathological features, little research has been conducted to explore the solid differences between both tumors.^9^, ^10^, ^11^ The research that has been done has also not delved into the specific molecular alterations of IVL. Here, we explored the detailed landscape of IVL by RNA‐sequencing, and compared its characteristics with that of LM.

## 2 METHODS

### 2.1 Tissue sample collection

Five female Chinese patients (median age 50 years, range 48‐54 years) were suspected to have IVL according to clinical history, physical, and imaging examinations (Figure 1), and their diagnoses were confirmed by pathologists after surgical operation. Contrast‐enhanced CT (Computed Tomography) revealed irregular uterine or parametrial masses in all patients, with long filling‐defects extending from the iliac vein or ovarian vein to the inferior vena cava and entering the right atrium. All patients were undergoing cardiopulmonary bypass‐assisted surgery, with deep hypothermia circulation arrest. The right atrium and inferior vena cava were opened, and the uterus, ovary, and tumor embolus were removed. Histopathological and immunohistochemical exam of the tumor confirmed IVL diagnosis (Figure S1). Another five female patients (median age 41.6 years, range 32‐50 years) who were diagnosed with LM were used as the control group. For each IVL/LM patient, tumor tissues (abbreviated as IVLT/LMT, with IVLT obtained from the tumor portion in the right atrium) and their morphologic normal nontumor tissues from the uterine myometrium (abbreviated as IVLN/LMN) were collected in the operating room immediately (≤15 minutes) after tissue removal and were snap frozen in liquid nitrogen and stored at −80°C until analysis was conducted. IVL tumors and paired adjacent nontumor Formalin fixed and paraffin embedded (FFPE) tissue samples were collected from surgically resected specimen from six IVL cases (five cases were described previously, and one additional case of a 45‐year‐old was added) to validate the sequencing results using RT‐qPCR. To guarantee suitable tissue quality and accuracy, the quality and classification of all collected tissues were confirmed under a microscope by two qualified pathologists.

### 2.2 Immunohistochemistry staining

Resected tissue specimens were fixed in 10% neutral formalin for 6‐24 hours, and processed by paraffin embedding. The paraffin‐embedded tissue blocks were then cut into 4 μm‐thick sections. The sections were dewaxed and rehydrated using established protocols, then washed with phosphate buffered saline (PBS) [catalog ZLI‐9062, ZSGB‐BIO, China] and antigen retrieved by microwave for 5 minutes in PBS solution. Slides were then blocked by turns with H2O2 and normal goat serum at room temperature for 15 minutes, followed by a 1‐hour (h) incubation at 37°C with primary antibodies against SMA [catalog AB32575, Abcam, Dilution 1:200], Desmin [catalog AB32362, Abcam, Dilution 1:1000], and CD10 [catalog ZM‐0283, ZSGB‐BIO, China, Dilution 1:100] respectively. Slides were then washed with PBS and incubated with the secondary antibody [catalog AB205718, Abcam, Dilution 1:2000], at room temperature for 1 hour. Slides were then washed with PBS, developed with diaminobenzidine (DAB), and counterstained with hematoxylin. Finally, slides were dehydrated, hyalinized, and mounted. Positive and negative controls for each primary antibody were included in each staining bath.

The staining was analyzed by two qualified pathologists independently, using a transmission light microscope (Olympus BX 41) under 200× magnification. The stained sections were scanned to virtual digital slides using a digital whole slide scanner (NanoZoomer‐XR, Hamamatsu, Japan), then viewed through NDPView 2.0 software (HAMAMATSU PHOTONICS). Slide images were exported as JPEG image files with internal bars (Figure S1).

### 2.3 RNA extraction and RT‐qPCR

Total RNA was extracted from fresh tissues using TRIzol (Invitrogen, 10296010) and from FFPE tissues using an RNeasy FFPE kit (QIAGEN, 73504) according to the manufacturer's protocol. RNA degradation and contamination were detected on 1% agarose gels. RNA purity was checked using the NanoPhotometer® spectrophotometer (IMPLEN). RNA concentration was assessed using Qubit® RNA Assay Kit in Qubit® 2.0 Flurometer (Life Technologies, Q33230). RNA integrity was examined by using an RNA Nano 6000 Assay Kit for the Bioanalyzer 2100 system (Agilent Technologies, 5065‐4401). Further, 2 μg of total RNA was used for PCR, and oligo(dT) was used for cDNA preparation. Real‐Time PCR was performed with Power SYBR Green PCR Master Mix (TransGen) on the ABI 7500 fast real‐time PCR system. The amplification reaction procedure was: 95°C for 10 minutes, followed by 95°C for 15 seconds and 60°C for 1 minute for 40 cycles. GAPDH was used as an internal control, and the relative expression level of mRNA was calculated by relative quantification (2^−△△CT^) method. The range of the obtained Ct values was 15‐29. Primer sequences are listed in Table 1.

**TABLE 1:** The primers used for qRT‐PCR with their sequence

Gene	Forward primer	Reverse primer
*CXCL8*	AACTGAGAGTGATTGAGAGTGG	ATGAATTCTCAGCCCTCTTCAA
*LIF*	CAACAACCTGGACAAGCTATG	GTACACGACTATGCGGTACAG
*SH2D2A*	GGTGCTACTTGGTGCGGTTCAG	CAGGAAGTGGCGGCAGCAAG
*GATA2*	AAGGCTCGTTCCTGTTCAGAAG	CCCATTCATCTTGTGGTAGAGG
*VASH2*	GAATGAAGATCCTGAAACCTGC	TCATTCAGAGTGCTCAGATCAG
*GAPDH*	GGCAGTGATGGCATGGACTGT	CCTTCATTGACCTCAACTACA

### 2.4 Library preparation for transcriptome sequencing

For the RNA sample preparation, 3 µg of RNA per sample was used as input material. According to the manufacturer's recommendations, the sequencing libraries were generated using NEBNext® UltraTM RNA Library Prep Kit for Illumina® (NEB, E7530L‐96 reactions), and index codes were added to attribute sequences to each sample. In general, mRNA was purified using poly‐T oligo‐attached magnetic beads. Then, under elevated temperature in NEBNext First Strand Synthesis Reaction Buffer(5×), fragmentation was carried out using divalent cations. The first strand cDNA was synthesized using random hexamer primer and M‐MuLV Reverse Transcriptase (RNase H‐NEB, M0253S), then the second strand cDNA synthesis was subsequently carried out using DNA Polymerase I and RNase H, and the remaining overhangs were converted into blunt ends via exonuclease/polymerase activities. NEBNext Adaptor with hairpin loop structure was ligated to adenylated 3’ends of DNA fragments to prepare for hybridization, and the library fragments were purified using AMPure XP system (Beckman Coulter). Then, 3 µL of USER Enzyme (NEB, M5505S) and adaptor‐ligated cDNA were incubated at 37°C for 15 minutes, followed by 5 minutes at 95°C before PCR. PCR was performed with Phusion High‐Fidelity DNA polymerase, Universal PCR primers, and Index (X) Primer. Finally, PCR products were purified via AMPure XP system and the library quality was assessed using the Agilent Bioanalyzer 2100 system.

### 2.5 Quantification of gene expression level

HTSeq v0.6.0 was used to count the number of reads mapped to each gene. Here, we used the Bayesian framework to avoid the normalization problem by working with the posterior distribution of the gene's true count.^13^ Fragments per kilobase of exon per million fragments mapped (FPKM) of each gene was calculated based on the length of the gene and reads count mapped. Cuffdiff (v2.1.1) was used to calculate FPKMs of both lncRNAs and coding genes in each sample.^14^

### 2.6 Differential expression analysis

**FIGURE 2:**
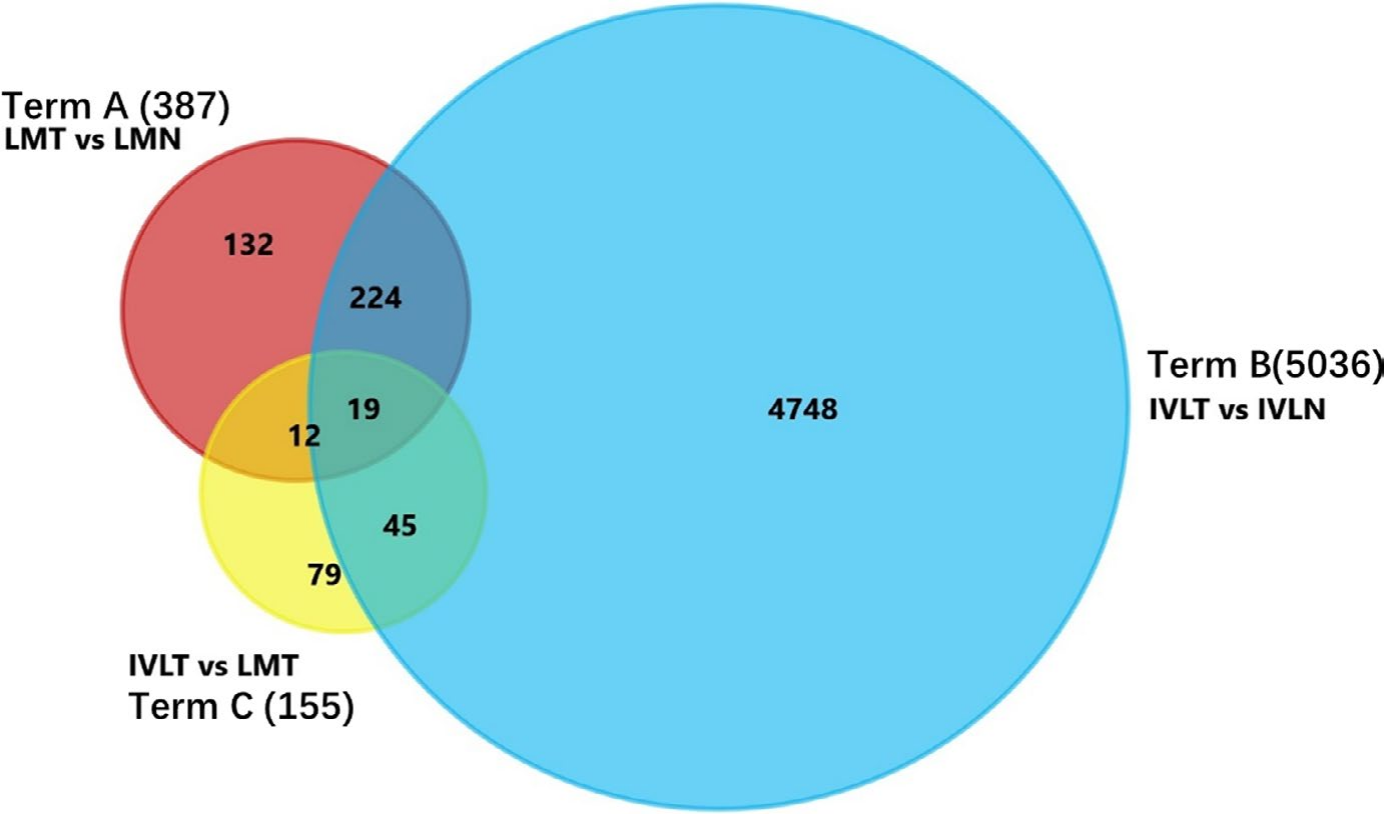
Venn diagram for DEGs in term A (LMT vs LMN), term B (IVLT vs IVLN) and term C (IVLT vs LMT). IVLT represents IVL tumor tissues; IVLN represents IVL‐adjacent normal tissues; LMT represents leiomyoma tumor tissues; and LMN represents leiomyoma‐adjacent normal tissues. The number in the circle represents the number of identified DEGs

The differential expression profile and analysis were performed in the following combinations: LM tissues and their adjacent normal tissues (term A); IVL tissues and their adjacent normal tissues (term B); and IVL tissues and LM tissues (term C) (Figure 2).

Differential expression patterns between groups were analyzed by the DESeq2 R package (1.10.1).^15^ DESeq2 provides statistical routines for estimating variance‐mean dependence and determining differential expression in count data from high‐throughput sequencing assays based on a model using the negative binomial distribution. To control for false discovery rate, *P*‐values were adjusted using the Benjamini and Hochberg approach. Genes with an adjusted *P*‐value < .05 as calculated by DESeq2 were qualified as differentially expressed genes (DEGs).

### 2.7 Enrichment and target analysis

The Disease Ontology (DO: http://www.disease‐ontology.org/), Gene Ontology (GO: http://geneontology.org/), KEGG (http://www.genome.jp/kegg/), and Reactome （https://reactome.org/） databases were used to perform enrichment analysis in order to annotate the DEGs within clinical and disease‐relevant data sets, and to explore other involved pathways. DO, GO, and Reactome terms with corrected *P*‐values < .05 were classified as being significantly enriched in the DEGs. The Cluster Profiler R package was used to test the statistical enrichment of DEGs in KEGG pathways.

We focused on angiogenesis and antiapoptosis pathways, which were two important pathways in tumor development and also may be involved in the different features between IVL and LM. DEGs and DE lncRNAs enriched in angiogenesis and antiapoptosis KEGG pathways, as well as DO, GO, and Reactome terms involved in these pathways, were collected and analyzed in the gene expression data.

### 2.8 Statistical analysis

Two‐sided Student's *t*‐test was performed to analyze the differences in expression using RT‐qPCR. Tukey's multiple comparison test was used to analyze the variance of the data and to estimate the level of significance. Log‐transformation was performed to approximate normal distribution of data before implementation of parametric tests. Statistical analysis was performed with SPSS19.0 software (USA). All data are presented as mean ± standard deviation of three independent experiments. *P*‐values < .05 were considered to be significantly different.

## 3 RESULTS

### 3.1 Differentially expressed genes

We identified 48 652 genes in all 20 sequencing samples including IVL tumor tissue, IVL‐adjacent normal tissues, LM tumor tissues and LM adjacent normal tissues. The DEG analyses were performed among these groups.

We compared LM tumor tissues and their adjacent normal tissues expression profile (term A), and identified 387 DEGs, including 124 upregulated DEGs and 263 downregulated DEGs. By comparing IVL tumor tissues and their adjacent normal tissues (term B), we identified 5036 DEGs in which 2220 genes were upregulated and 2816 downregulated in IVL tumor tissues. We also compared IVL tumor tissues to LM tumor tissue expression profile (term C), and identified 155 DEGs (Figure 2), with 103 DEGs upregulated and 52 downregulated in IVL tumor tissues. The volcano plot shows there were much more genes that were not significantly changed (adjusted *P*‐value > .05, the common thresholds for DEG identification) (Figure S2).

Further common differential gene (co‐DEGs) analyses were carried out. Two hundred and forty three DEGs were identified in common between term A and term B, while 31 co‐DEGs between term A and term C, 64 co‐DEGs between term B and term C, and 19 co‐DEGs of term A, B and C. In 31 co‐DEGs between terms A and C, 12 genes did not correlate with term B. Similarly, of the 64 c‐DEGs between terms B and C, 45 did not belong to term A (Figure 2).

**FIGURE 3:**
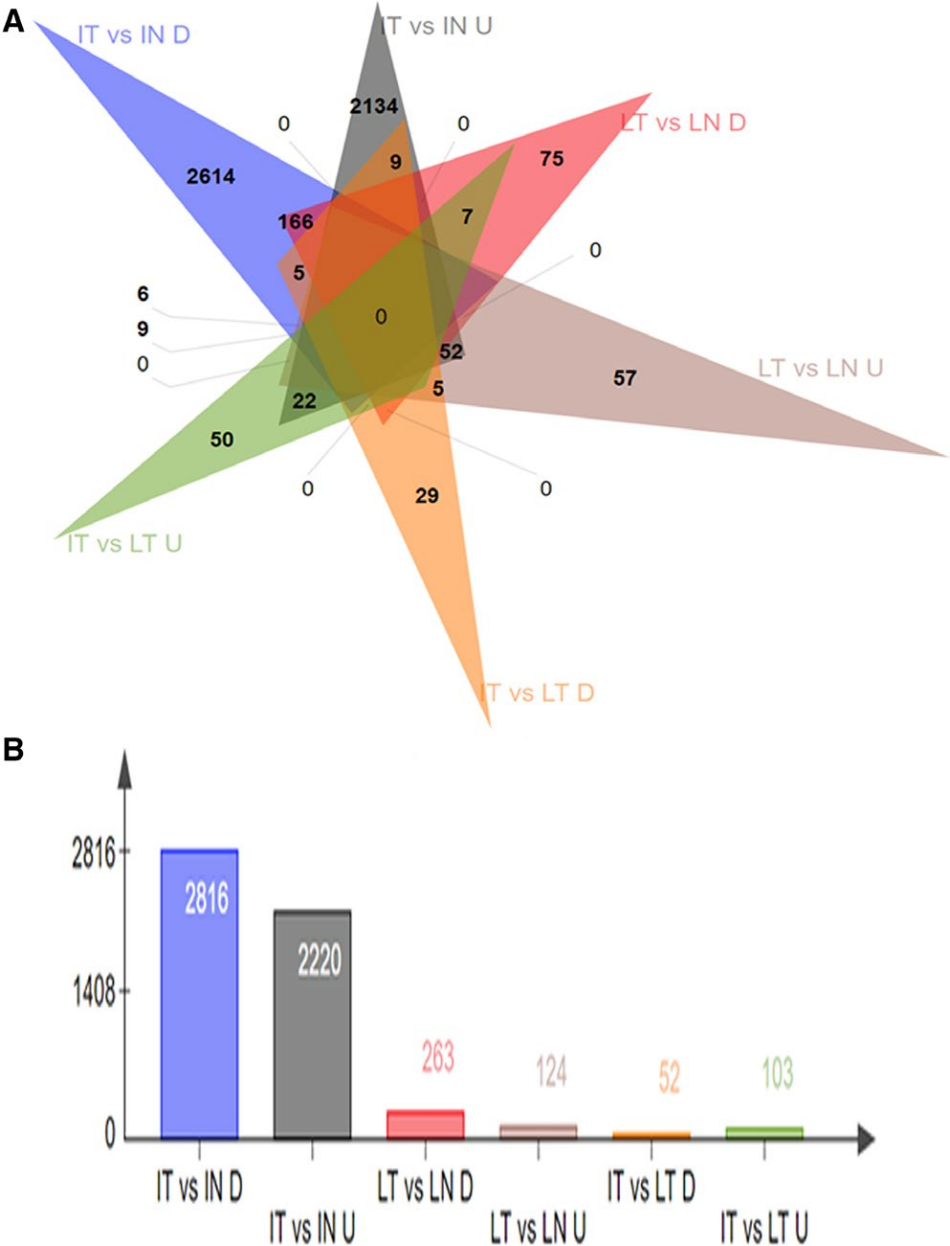
Venn diagram for up/downregulated DEGs in term A (LMT vs LMN), term B (IVLT vs IVLN) and term C (IVLT vs LMT). (A) Venn diagram for DEGs in terms A, B, and C where IT represents IVL tumor tissues; IN represents IVL‐adjacent normal tissues; LT represents leiomyoma tumor tissues; LN represents leiomyoma‐adjacent normal tissues; U represents upregulated DEGs; and D represents downregulated DEGs. The number in the triangle represents the number of DEGs. B: Quantification of Venn diagram for up/downregulated DEGs

The ratio of upregulated to downregulated genes of the 5259 total DEGs (terms A, B and C combined) were analyzed separately as shown in Figure 3. In term A, there were approximately two times as many downregulated DEGs as there were upregulated DEGs. Term B also had more downregulated DEGs than upregulated DEGs, though the ratio was dissimilar to that of term A. This trend is opposite in term C, where there were nearly two times as many upregulated DEGs as there were downregulated DEGs.

Hierarchical clustering for 20 libraries showed that the expression profile for IVL tumor tissue and adjacent normal tissues were different from that of LM tumor tissue (Figure S3). The IVL tumor expression profiles were relatively concentrated, while the LM tumor expression profiles were relatively dispersed (Figure S3). These results strongly indicated that the gene expression profile of IVL was different from LM.

### 3.2 GO enrichment analysis

There were 689, 774, and 199 GO terms significantly enriched in terms A, B, and C, respectively. The significantly up‐ and down‐regulated GO terms are shown in Table 2, Figures S4 and S8‐S11. The 199 significantly enriched GO terms in Term C were focused on for further analysis, to differentiate IVL and LM (Figure S4). Of these 199 GO terms, no molecular function terms were enriched. Of the biological process terms, the most significantly enriched terms were “Positive regulation of cytokine secretion,” “regulation of ERK1 and ERK2 cascade,” and “ERK1 and ERK2 cascade.” Of the biological process terms, the term that contained the most DEGs was “Protein secretion”.

**TABLE 2:** Significantly enriched GO terms

Terms	A	B	C	D
All	689	774	199	259
Up‐regulated	1025	180	174	24
Down‐regulated	109	1142	17	348

### 3.3 DO enrichment analysis

A total of 442 DO terms were significantly enriched in our study. There were 25, 175, and 96 DO terms significantly enriched in terms A, B and C, respectively (Table 3, Figures S5 and S9). In term C, primary bacterial infectious disease, tuberculosis, uterine disease, arteriosclerosis, and bacterial infectious disease were the most significantly enriched DO terms (Figure S5).

**TABLE 3:** Significantly enriched DO terms

Terms	A	B	C	D
ALL	25	175	96	146
Up‐regulated	123	3	151	0
Down‐regulated	0	293	1	137

### 3.4 KEGG pathway analysis

There were five, 19, and five KEGG pathways significantly enriched in terms A, B and C, respectively (Table 4, Figures S6 and S10). By comparing the data from IVL tumor and LM tumor samples, five pathways were identified as being significantly enriched, including *Wnt* signaling, Focal adhesion, Gap junction, Rheumatoid arthritis, and Amoebiasis pathways. The enriched pathways were shown in Figure S6.

**TABLE 4:** Significantly enriched KEGG pathways

Terms	A	B	C	D
All	5	19	5	5
Up‐regulated	12	16	2	5
Down‐regulated	9	17	6	13

### 3.5 Reactome enrichment analysis

There were 15, 20, and eight reactome categories significantly enriched for in terms A, B and C, respectively (Table 5, Figures S7 and S11). Within term C, eight categories were significantly enriched. While no upregulated categories were found, 18 downregulated categories were identified in our study (Figure S7).

**TABLE 5:** Significantly enriched reactome categories

Terms	A	B	C	D
All	15	20	8	2
Up‐regulated	23	37	0	4
Down‐regulated	20	19	18	6

Overall, several enrichment analyses identified differentially expressed categories in term C, which suggests multiple pathological and biological processes that differentiate IVL from LM. Key pathways involved in IVL pathological processes may be apparent in these categories, such as angiogenesis and antiapoptosis pathways.

### 3.6 Angiogenesis‐related pathways and gene analysis

**FIGURE 4:**
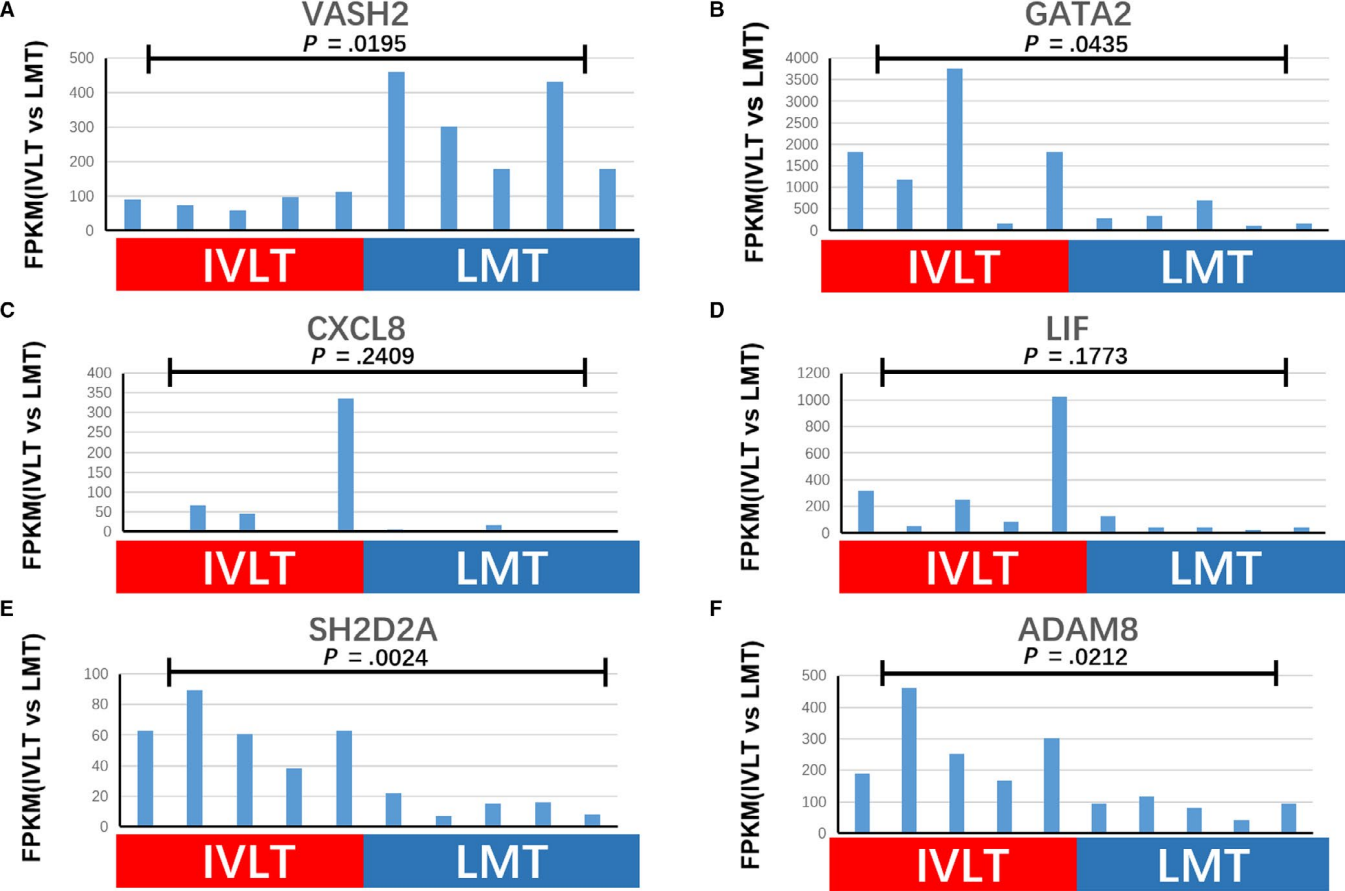
Angiogenesis factors enriched in IVL tumors. FPKM values for angiogenesis factors enriched in IVL tumors, including *VASH2, GATA2, CXCL8, LIF, SH2D2A*, and *ADAM8*. Student's *t*‐test was used to analyze the differences between the two groups and to estimate the level of significance. Log‐transformation was performed to approximate normal distribution of data before implementation of parametric tests

To further understand the relevance of different angiogenesis and apoptosis‐related pathways in IVL vs LM, we first identified angiogenesis‐related terms that were significantly enriched in the GO analysis for term C. These terms were GO:0045765 (regulation of angiogenesis), GO:0001525 (angiogenesis), and GO:0045766 (positive regulation of angiogenesis). Key genes related to these pathways, such as *SH2D2A* (SH2 Domain Containing 2A), *VASH2* (Vasohibin 2), *ADAM8* (ADAM Metallopeptidase Domain 8), *GATA2* (GATA Binding Protein 2), *CXCL8* (C‐X‐C Motif Chemokine Ligand 8), and *LIF* (Leukemia Inhibitory Factor), were analyzed in our research. *VASH2* was significantly downregulated (*P* = .0195) in IVL *SH2D2A, ADAM8* and *GATA2* were significantly upregulated (*P* = .0024, *P* = .0212, and *P* = .0435, respectively) in IVL *CXCL8* and *LIF* were also upregulated in IVL but were not statistically different (*P* = .2409 and *P* = .1773, respectively) (Figure 4).

### 3.7 Apoptosis‐related pathways and gene analysis

**FIGURE 5:**
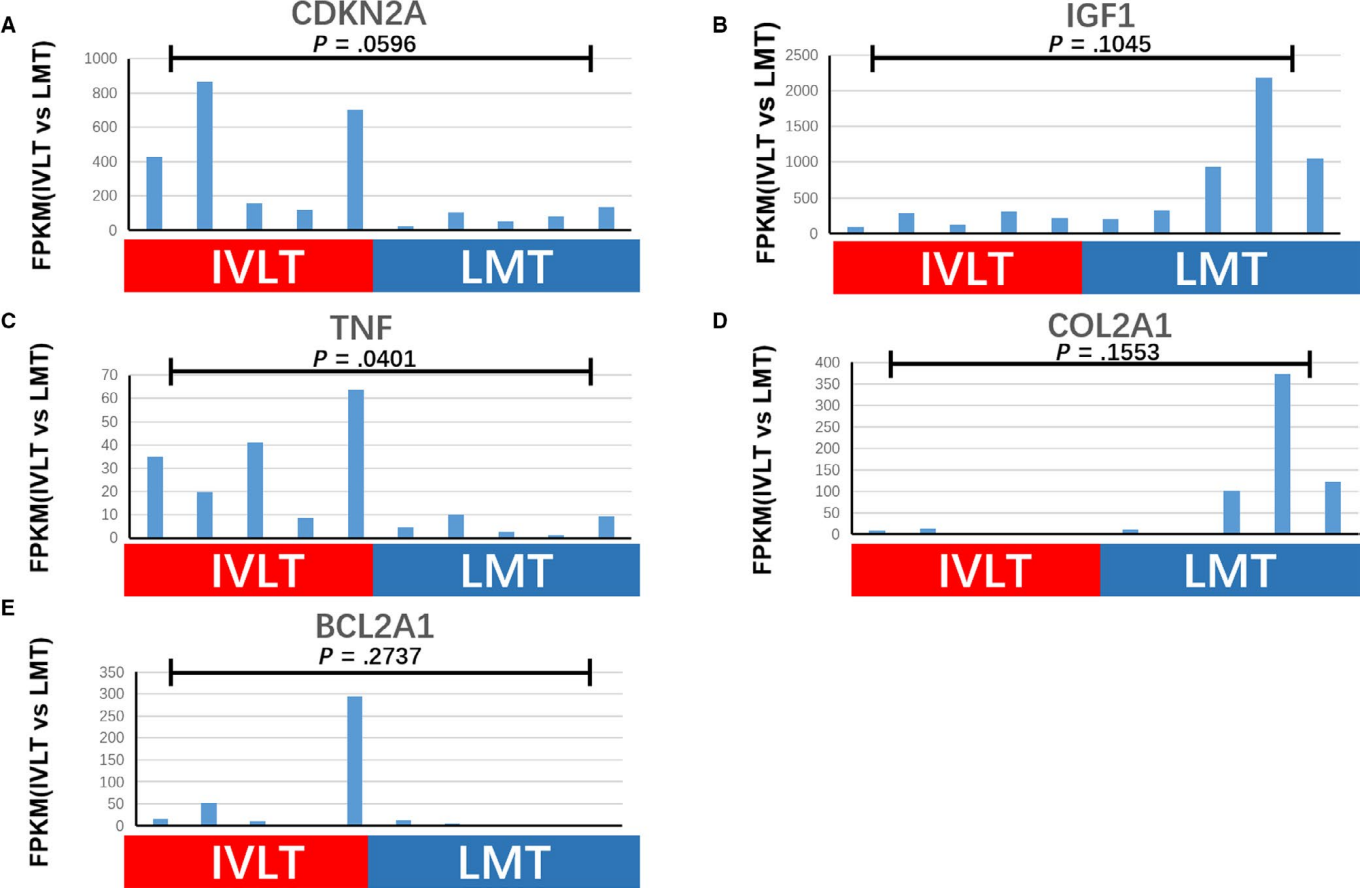
Apoptosis factors enriched in IVL tumors. FPKM values for apoptosis factors enriched in IVL tumors, including *CDKN2A, IGF1, TNF, COL2A1*, and *BCL2A1*. Tukey's multiple comparison test was used to analyze the differences among 10 samples from two groups and to estimate the level of significance

A similar analysis was conducted to look at apoptosis‐related pathways in term C. GO:2001234 (negative regulation of apoptotic signaling pathway), GO:2001233 (regulation of apoptotic signaling pathway), GO:0 034 390 (smooth muscle cell apoptotic process), and GO:0034391 (regulation of smooth muscle cell apoptotic process) were significantly enriched, and 11 key genes were identified. Of these, *CDKN2A* (Cyclin Dependent Kinase Inhibitor 2A), *TNF* (Tumor Necrosis Factor), and *BCL2A1* (BCL2 Related Protein A1) were upregulated, though only *TNF* showed a statistical difference (*P* = .0401). While *COL2A1* (Collagen Type II Alpha 1 Chain) and *IGF1* (Insulin Like Growth Factor 1) were both downregulated in IVL, neither showed a statistical difference (*P* = .1553 and *P* = .1045 respectively) (Figure 5).

### 3.8 Other enriched pathways and lncRNAs

Degradation of the extracellular matrix and components of cell adhesion are important factors for tumor migration. In the reactome analysis in term C, we identified degradation of the extracellular matrix (ID:1474228) and ECM (extracellular matrix) proteoglycans (ID:3000178) terms as being enriched (Figure S7). Furthermore, in the KEGG enrichment analysis, we identified hsa04310 (*Wnt* signaling pathway) and hsa04510 (Focal adhesion) as being key pathways in this term (Figure S6). These pathways are also involved in tumor development.

In the DO enrichment analysis, we identified several pathways involved in arteriosclerotic cardiovascular disease (DOID:2348), vasculitis (DOID:865), uterine disease (DOID:345), and endometriosis of the uterus (DOID:288) (Figure S5).

**FIGURE 6:**
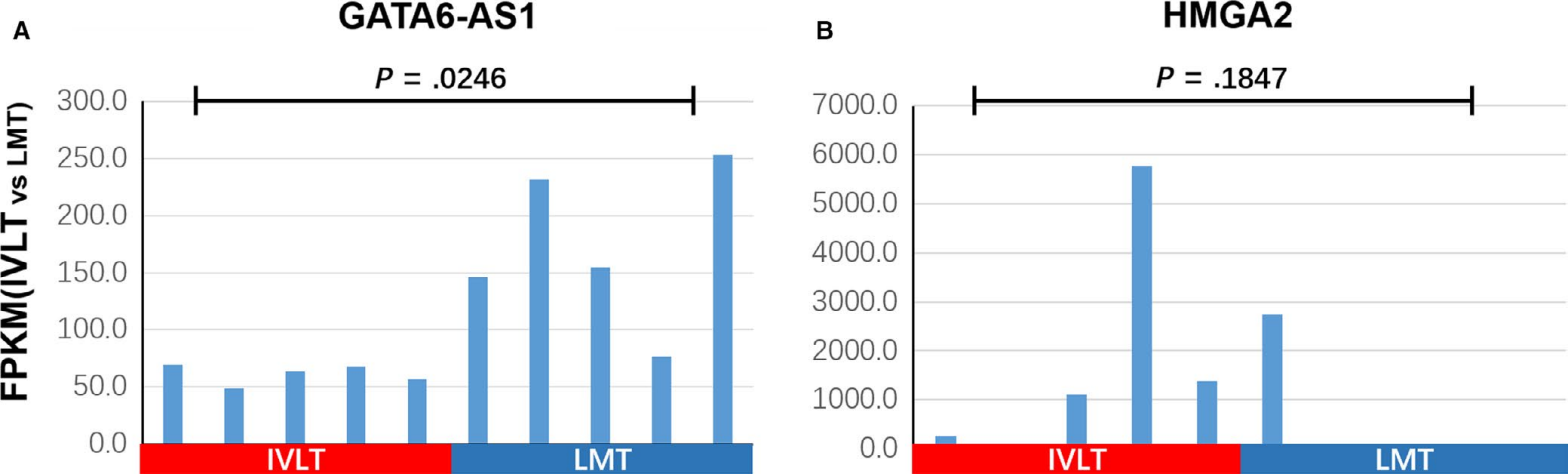
Comparison of expression pattern of lncRNAs *GATA6‐AS1* and *HGMA2* in IVLT and LMT samples. Comparison of expression pattern of lncRNAs *GATA6‐AS1* and *HGMA2* in IVL and LM tumor samples. Tukey's multiple comparison test was used to analyze the differences among 10 samples from two groups and to estimate the level of significance

We also identified the lncRNAs *GATA6‐AS1* (GATA6 Antisense RNA 1) and *HMGA2* (High Mobility Group AT‐Hook 2) within term C. In our data, *GATA6‐AS1* was significantly downregulated in IVL tumor samples (*P* = .0246) (Figure 6A). *HMGA2* was upregulated in 4/5 cases of IVL, but showed no statistical difference comparing to LMT (*P* = .1847) (Figure 6B).

### 3.9 Rt‐qPCR validation of DEGs in IVL tissue

**FIGURE 7:**
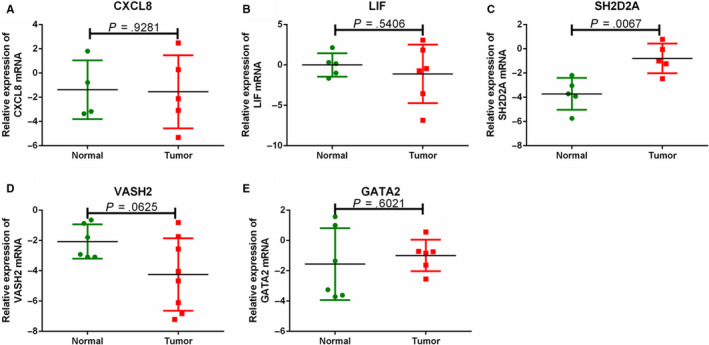
RT‐qPCR validation of selected DEGs in Chinese IVL Patients. RT‐qPCR validation of selected DEGs in IVL patient samples, including *CXCL8, LIF, SH2D2A, VASH2,* and *GATA2*. Tukey's multiple comparison test was used to analyze the differences from two groups and to estimate the level of significance. Log‐transformation was performed to approximate normal distribution of data before implementation of parametric tests

To further verify the expression of *CXCL8, LIF, SH2D2A, VASH2,* and *GATA2* in IVL tissues, we analyzed six (five previously presented and one additional) Chinese IVL cases by RT‐qPCR. The results showed that the expression of *SH2D2A and GATA2* were upregulated (*P* = .0067 and *P* = .6021, respectively), while *CXCL8, LIF*, *and VASH2* were downregulated in IVL tumor samples compared to nontumor tissue (*P* = .9281, *P* = .5406 and *P* = .0625, respectively) (Figure 7). There was no statistical difference between the expression levels for most of these genes except *SH2D2A*, possibly due to the low sample number and specimen sample procedure (FFPE sample may cause molecule alterations in tissue).

## 4 DISCUSSION

Intravenous leiomyomatosis (IVL) is a rare entity characterized by intravascular growth with histologically benign smooth muscle cells.^1^ The accuracy of diagnosis of IVL is mainly limited by the lack of characteristic image features and specific biomarkers. In addition to this, the rare incidence of IVL makes it difficult to diagnose prior to surgery; therefore, the collection of fresh tissue samples for research is limited. There are only a few case reports and experimental studies on IVL.^16^, ^17^ In the field of genetics research, Boston research teams have conducted a karyotype analysis of two cases of IVL and found that both cases had genetic abnormalities of der(14)t(12; 14)(q15; q24).^11^ In 2014, a research team from Yale University used the comparative genomic hybridization (CGH) method to study nine cases of IVL and found a series of copy number variations.^18^ Even with these advances, little is known about the molecular mechanisms of this cancer. Here, we provide a detailed landscape of IVL in comparison to a similar entity, uterine leiomyoma (LM), using RNA‐sequencing methods.

Based on clinical and pathological analysis, we found that IVL presented as a quite different entity from LM in several aspects, such as tumor extension, neovascular genesis, and recur.^4^ In this study, we focused on the gene expression profile of IVL, and the differential expression patterns from LM. We carried out high‐throughput RNA‐sequencing on tumor and normal samples from five IVL patients and five LM patients and compared the differences in gene expression. From a whole landscape view, 5036 differentially expressed genes (DEGs) for IVL tumors and tumor‐adjacent tissue were identified, while only 387 DEGs were identified for LM tumor and tumor‐adjacent tissue (Figures 2 and 3). Our results indicated that the gene expression spectrum of IVL was more complex than LM. Furthermore, there were 155 DEGs that were identified by comparing the expression profile of IVL tumors to LM tumors, with IVL tumor expression profiles being more concentrated than LM tumor expression profiles (Figure 2, Figure S3). All these data suggest that IVL is genetically quite different from LM.

Angiogenesis is an important pathophysiological and tumorigenic process and is thought to be beneficial to the unique intravenous growth of IVL.^4^ High levels of proangiogenic factors are expressed in many kinds of tumor tissue. For example, *GATA2* regulates the development and proliferation of hematopoietic cells.^19^ *LIF* is involved in the induction of hematopoietic differentiation.^20^ *CXCL8* is a potent angiogenesis factor, as well as a major mediator of the inflammatory response.^21^ *SH2D2A* plays an important role in normal and pathological angiogenesis.^22^ Additionally, besides proangiogenesis factors, *ADAM8* may be involved in cell adhesion.^23^ In breast cancer, *ADAM8* promoted tumor dissemination and metastasis.^24^ All of these factors were upregulated in IVL tumors as compared to LM tumors, suggesting an increase in angiogenesis and tumor dissemination. Additionally, *VASH2*, an endogenous angiogenesis inhibitive factor,^25^ was downregulated in IVL tumor samples when compared to LM tumors. Interestingly, the lncRNA *GATA6‐AS1* was also downregulated in IVL *GATA6‐AS1* is involved in vascular disease and may be associated with the upregulation of *GATA2*. Using RT‐qPCR, we validated these results in samples from IVL patients and showed that the expression of *SH2D2A* and *GATA2* were upregulated, while *CXCL8, LIF*, and *VASH2* were downregulated in the IVL tumor samples compared to the nontumor samples (Figure 7). Compared with the sequencing data, four of five selected DEGs showed a similar trend. However, there was no statistical difference in these genes except *SH2D2A*, possibly due to the limited sample number and large individual differences. Altogether, IVL tumors seem to have higher expression level of angiogenesis factors when compared to LM tumors. Our result suggested that IVL tumors have the strong ability to promote angiogenesis for their unique growth patterns, further differentiating IVL tumors from LM tumors.

Negative regulation of apoptosis can inhibit cell death signaling pathways, promoting tumors to evade cell death and develop drug resistance.^26^ Many families of proteins, such as *IAPs* and *Bcl‐2* proteins, inhibit apoptosis by acting as negative regulators, while some factors, such as *cFLIP, BNIP3, FADD, Akt* and *NF‐κB,* promote apoptosis. Within the antiapoptosis signaling pathways identified in our analysis, *TNF* and *CDKN2A* induce cycle arrest and apoptosis.^27^, ^28^ *BCL2A1* reduces the release of proapoptotic cytochrome c from the mitochondria, thus blocking caspase activation and the induction of apoptosis.^29^ In previous a research conducted by our team, Bcl‐2 was found to be highly expressed in IVL, which is consistent with our present data.^4^ All the above three genes were upregulated in our IVL tumor cases. We also found that IVL tumors showed higher expression levels of antiapoptosis factors than LM tumors, which may be associated with the characteristic continuous extension growth and proliferation rate of IVL. The high expression of antiapoptosis factors of IVL also shows a marked difference from LM.

Components of our results are consistent with previous studies on IVL. Ordulu et al revealed that seven of 12 (58%) IVL cases expressed high levels of *HMGA2* by immunohistochemistry staining.^1^ Dal Cin P et al found that some cases of IVL presented the balanced translocation (12;14)(q14‐15;q23‐24), which is a commonly observed rearrangement in uterine myomas.^12^ *HMGA2* is located on 12q14.3, where chromosomal rearrangements frequently occur in IVL.^10^ *HMGA2* is involved in transcriptional regulation, and affects cell differentiation and proliferation.^31^, ^32^ The breakpoint on 12q14‐15 typically falls 5' to *HMGIC* (*HMGA2*) and results in aberrant expression of this gene. Dysregulation of the nonhistone chromatin factor *HMGA2* is associated with a potential pathogenetic mechanism in IVL.^30^ *HMGA2* also plays a role in normal mesenchymal growth in mice.^33^ The misexpression of *HMGA2* in a differentiated mesenchymal cell can cause mesenchymal tumorigenesis, even when *HMGA2* is a truncated version. In our data, *HMGA2* was upregulated in only 4/5 cases of IVL (Figure 7). However, in most of the LM tumor samples, and in normal tissue adjacent to IVL, expression levels of *HMGA2* were less than 1% of the expression in the IVL tumors. The different expression levels between IVL and LM tumors may therefore also suggest the distinct molecular basis underlying their diverse growth patterns.

There are still several limitations in the execution and interpretation of this study. Though part of our results were consistent with previous work, the system error from high‐throughput sequencing were hard to correct for and often lead to false positive results. We utilized RT‐qPCR to validate the RNA‐seq results, partially alleviating these concerns. Furthermore, inter‐ and intra‐tumoral heterogeneity can make analyses involving the identification of differential expression of genes difficult. Errors as a result of tumor heterogeneity can be corrected for by increasing sample size, though this may have not been completely corrected for in our data. This is also limited by the collection of numerous adequate samples, which is difficult due to the rare incidence of this disease. As the sample size increases, further verification and functional studies will be needed to validate these findings.

In summary, we compared the whole transcriptome data of IVL with LM and showed that the gene expression profile of IVL is more complex than LM. Enrichment analyses identified antiapoptosis and angiogenesis‐related pathways, including some key genes, that may partly explain the underlying molecular mechanism of the differences between IVL and LM. Based on this evidence, we argue that IVL is quite different from LM on molecular and genetic basis, as well as by morphologic and clinical characteristics, and may be qualified as a solid entity with uncertain malignant potential, rather than a unique variant of LM. Our findings, as well as further studies based on more samples, may help to refine the current WHO classification. Additionally, further detailed investigation may show that certain related DEGs—in particular, antiapoptosis‐related genes *CDKN2A* and *BCL2A1*, as well as angiogenesis‐related gene *CXCL8*—may be novel specific biomarkers for IVL.

## AUTHOR CONTRIBUTIONS

Wenze Wang and Guotao Ma designed the research. Wenze Wang and Yanfeng Wang analyzed the data and performed the statistical analyses. Yanfeng Wang, Fei Chen, Ming Zhang, Rujing Jia, Xingrong Liu, Chaoji Zhang, Jiang Shao, Zhiyong Liang, and Ninghai Cheng supervised the study. Wenze Wang wrote the first draft and Guotao Ma, Zhaohui Zhu, Qi Miao, and Zhiyong Liang made a critical revision of the manuscript for important intellectual content, and Guotao Ma had primary responsibility.

This study was approved by the Research Ethics Committee of Peking Union Medical College Hospital (S‐K509), and informed consent was obtained from each patient.

## Supporting information

Fig S1‐S11

## Data Availability

We are very supportive of uploading sequencing data to public databases to share data. After further digging deeper into the data and doing more work in the near future, we will be glad to upload the original sequencing data to the public database to share with everyone interested.
