# Supplementary material for: Intravenous leiomyomatosis is inclined to a solid entity different from uterine leiomyoma based on RNA‐seq analysis with RT‐qPCR validation
Source: Cancer Med. 2020 May 5;9(13):4581–92. doi: 10.1002/cam4.3098 (PMC7333852; doi:10.1002/cam4.3098)
Supplement: Supplementary file 1 — Fig S1‐S11 [file CAM4-9-4581-s001.docx]

# Supplements material


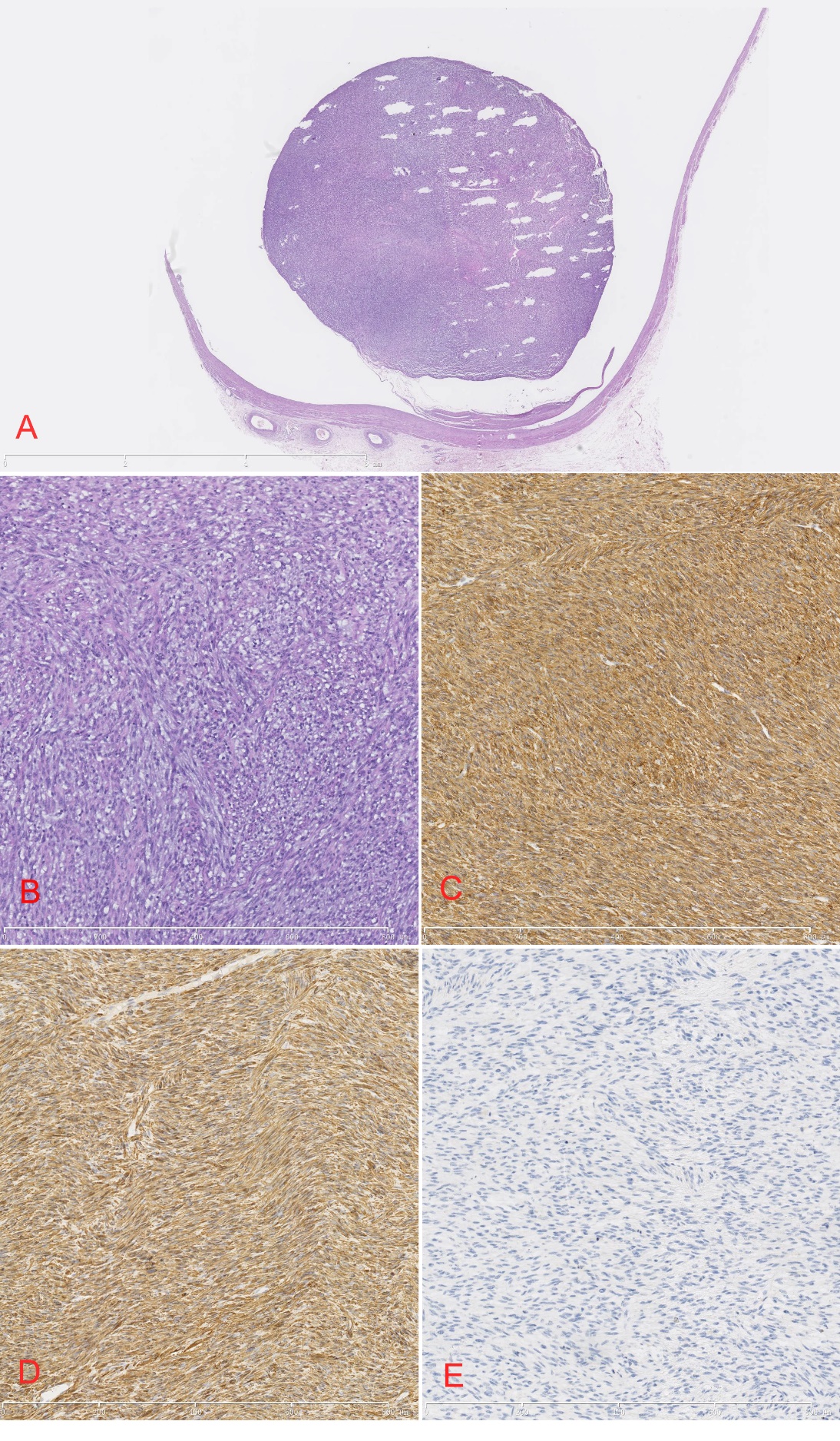


**Supplements 1. Histopathologic confirmation of IVL. A Low power view of IVL, tumor embolus (central) is encircled by the large venous wall (peripheral, partially damaged during the section preparation. B H&E staining shows the bland spindle cells. The characteristic immunohistochemical stainings are shown in C (SMA), D (Desmin) and E (CD10).**


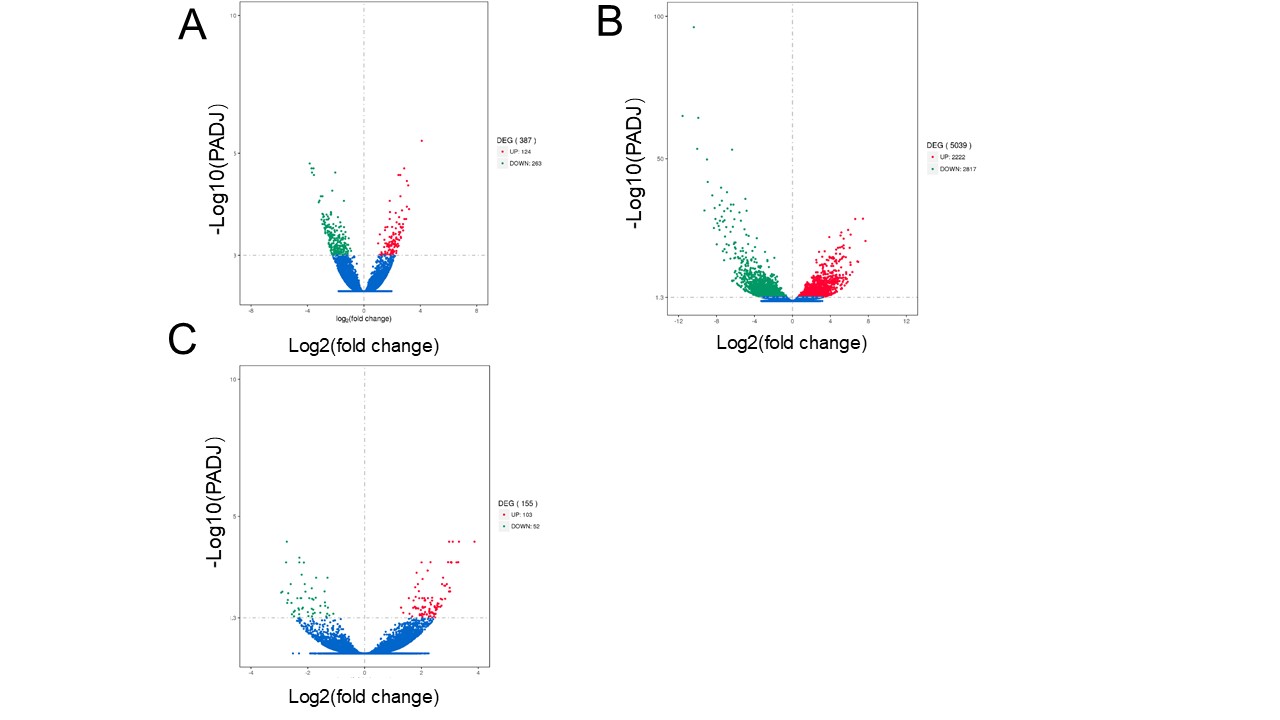


**Supplements 2. Volcano Plot of the DEGs in four terms.**

The red points in the plot represent the down-regulated mRNAs with statistical significance. The green points in the plot represent the up-regulated mRNAs.

A: LM tissues and their adjacent normal tissues (A term).

B: IVL tissues and their adjacent normal tissues (B term);

C: IVL tissues and LM tissue (C term);


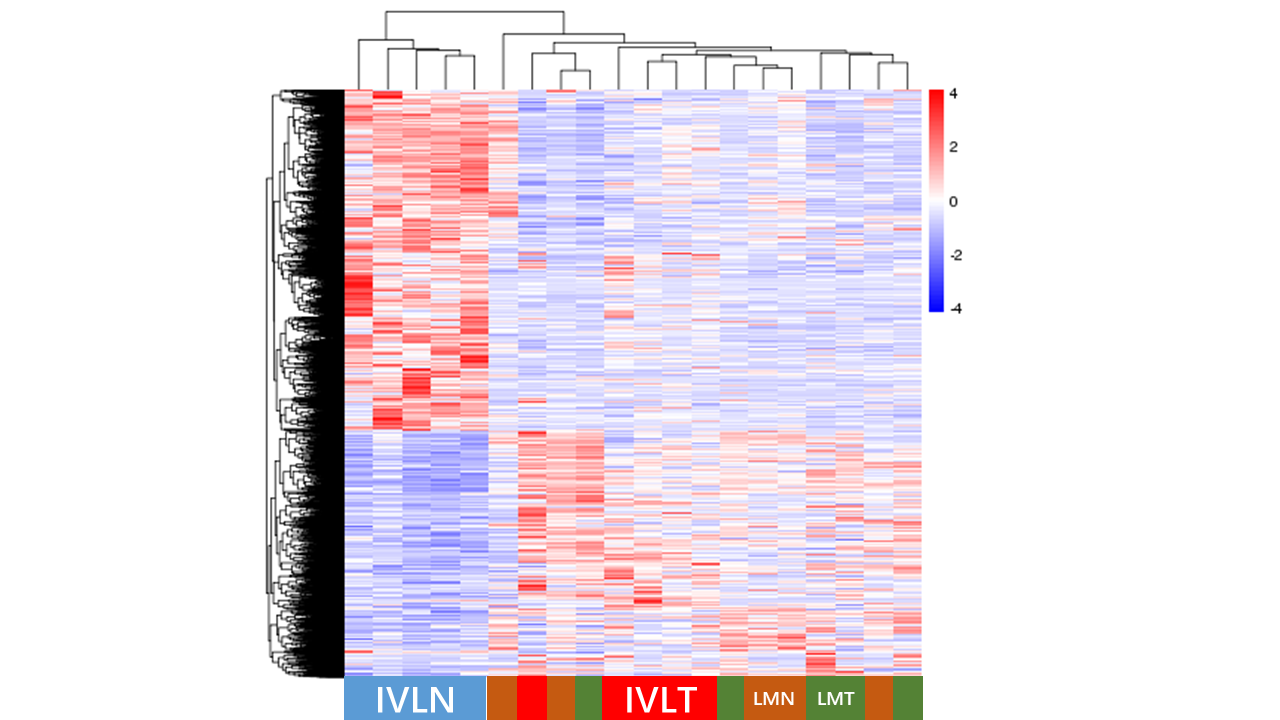


**Supplements 3. Hierarchical Clustering for 20 libraries obtained from IVL and LM patients.**

The blue block in the bottom stand for 5 IVL normal tissue samples;

The red block in the bottom stand for 5 IVL tumor tissue samples;

The brown block in the bottom stand for 5 LM normal tissue samples;

The green block in the bottom stand for 5 LM tumor tissue samples.


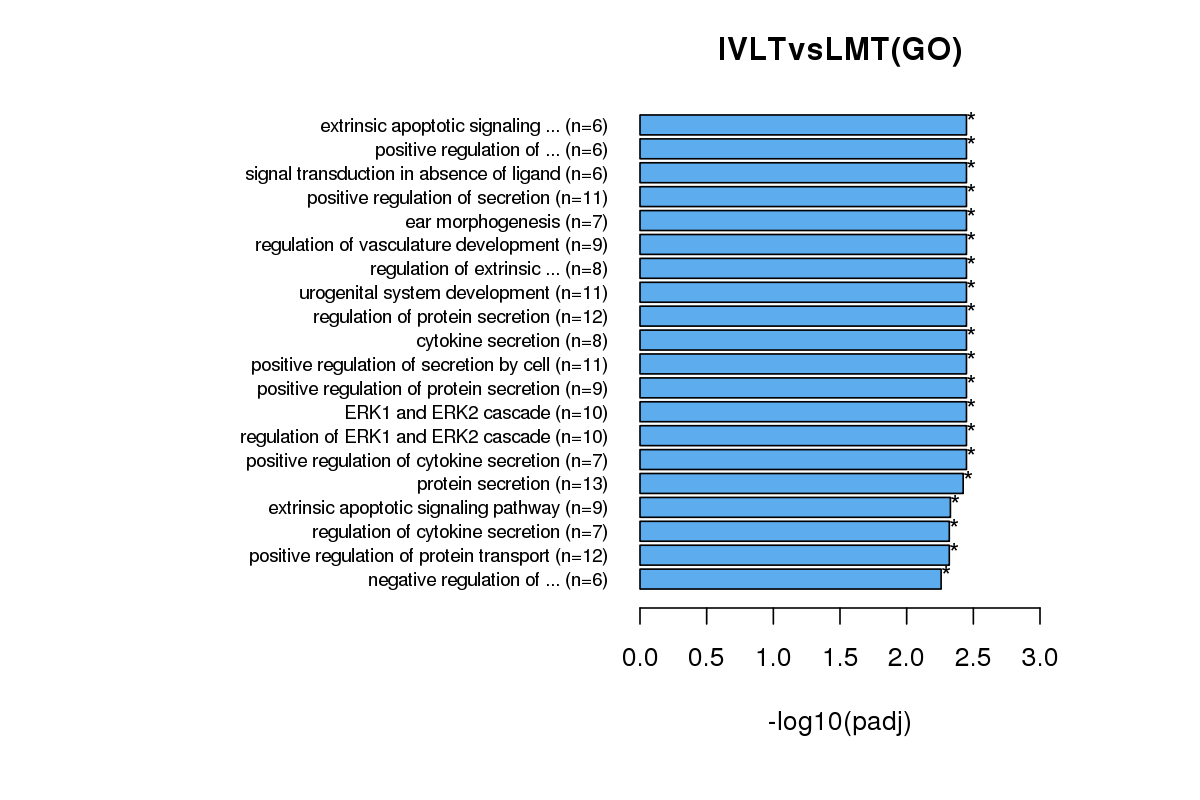


**Supplements 4. GO enrichment analysis of tumor tissues samples between IVL and LM (term C)**

Top 20 enrichment terms in GO analysis


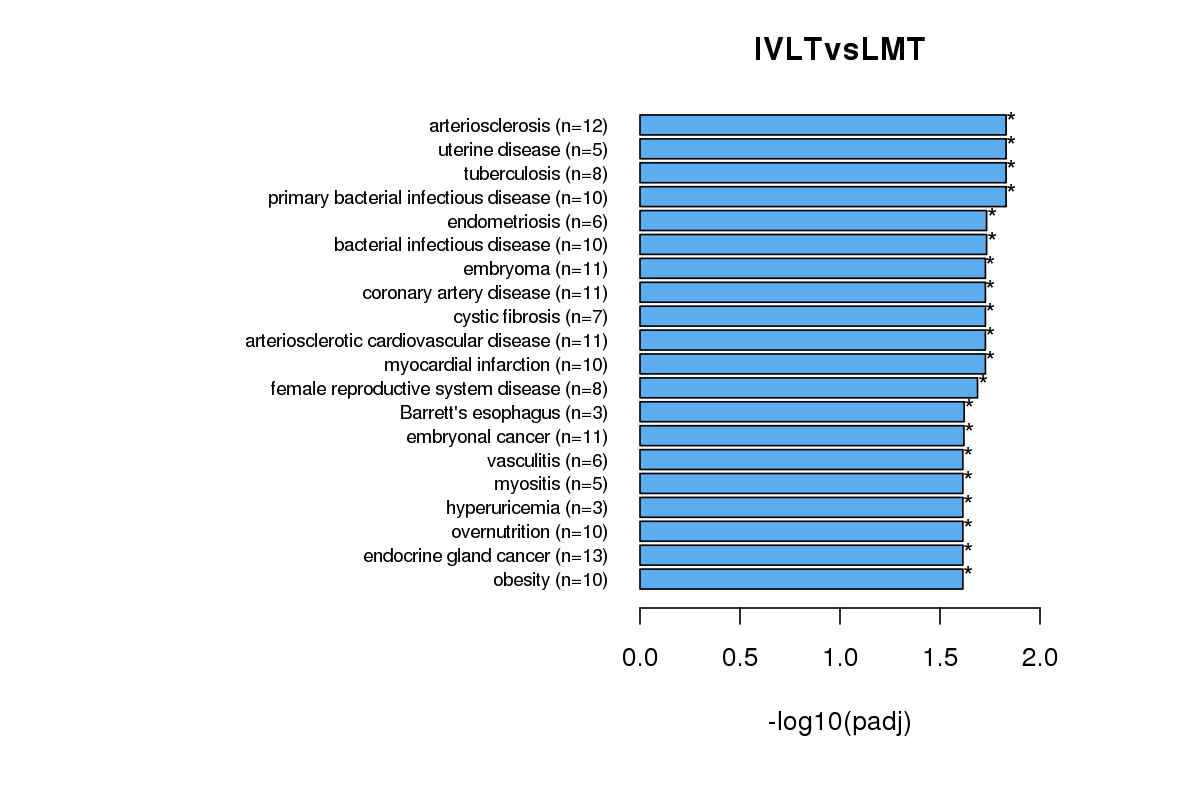


**Supplements 5. DO enrichment analysis of tumor tissues samples between IVL and LM (term C)**

Top 20 enrichment terms in DO analysis


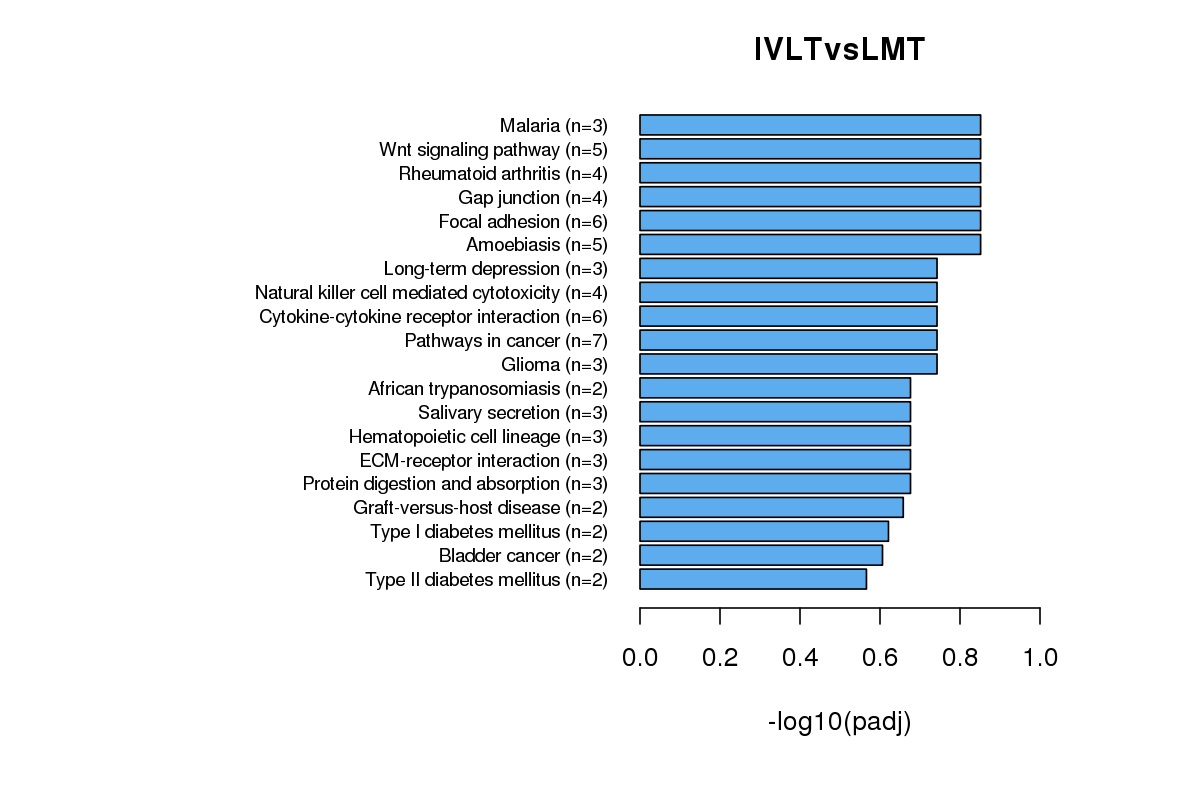


**Supplements 6. KEGG pathway analysis of tumor tissues samples between IVL and LM (term C)**

Top 20 enrichment pathways in KEGG analysis


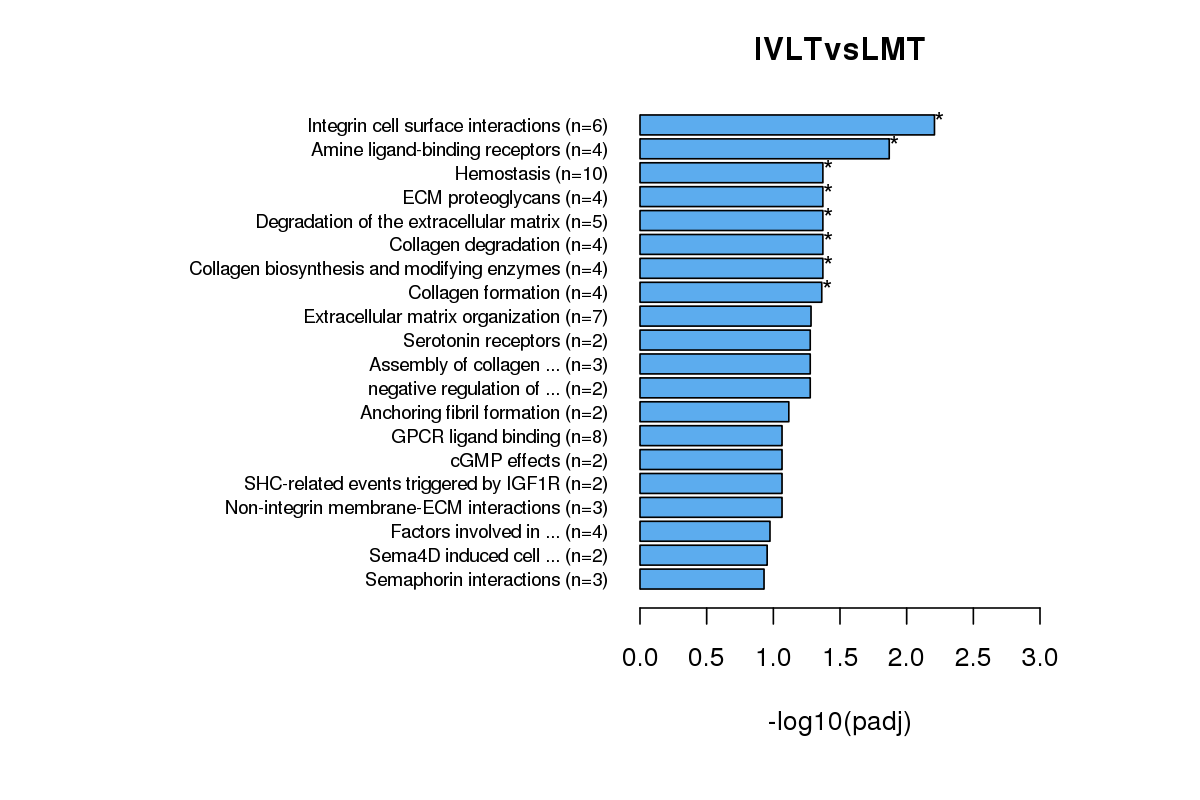


**Supplements 7. Reactome enrichment analysis of tumor tissues samples between IVL and LM (term C)**

Top 20 enrichment categories in reactome analysis


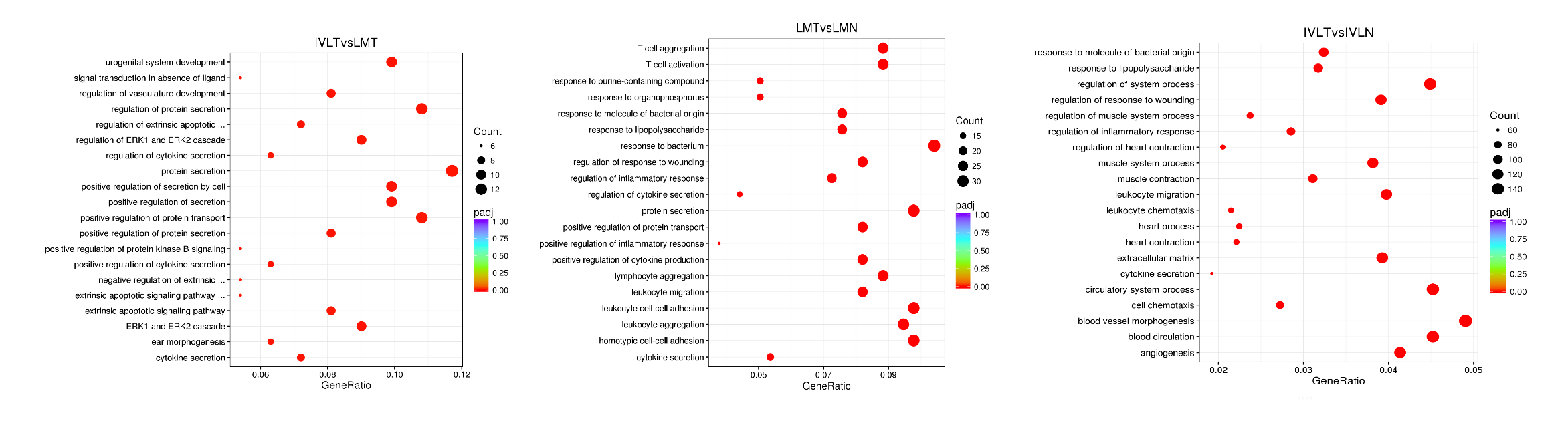


**Supplements** 8. **Plots for GO enrichment analysis of term A, B and C (from left to right).**

##
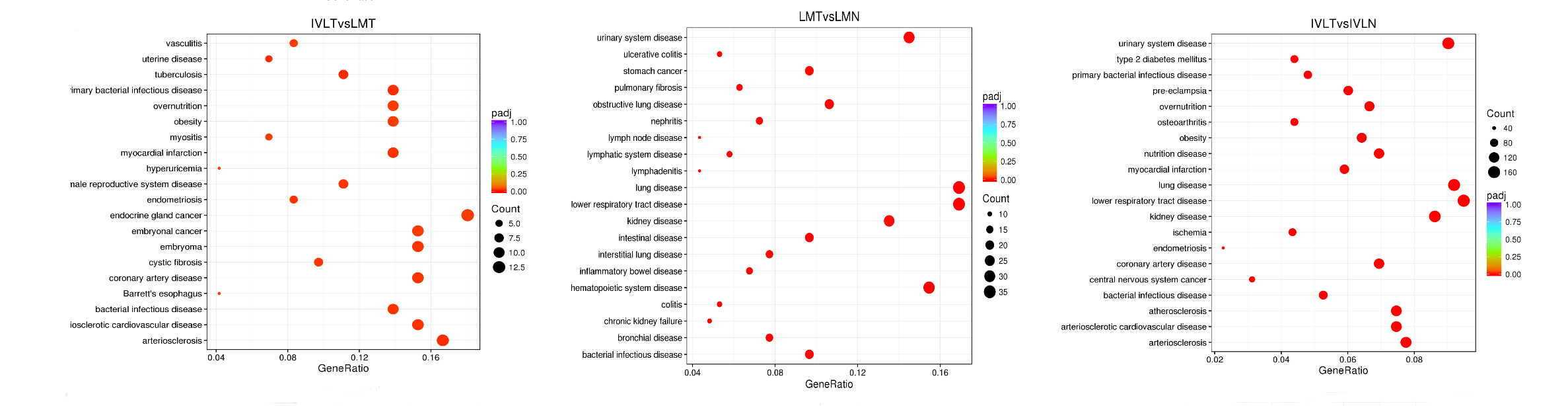


**Supplements** 9. **Plots for DO enrichment analysis of term A, B and C (from left to right).**


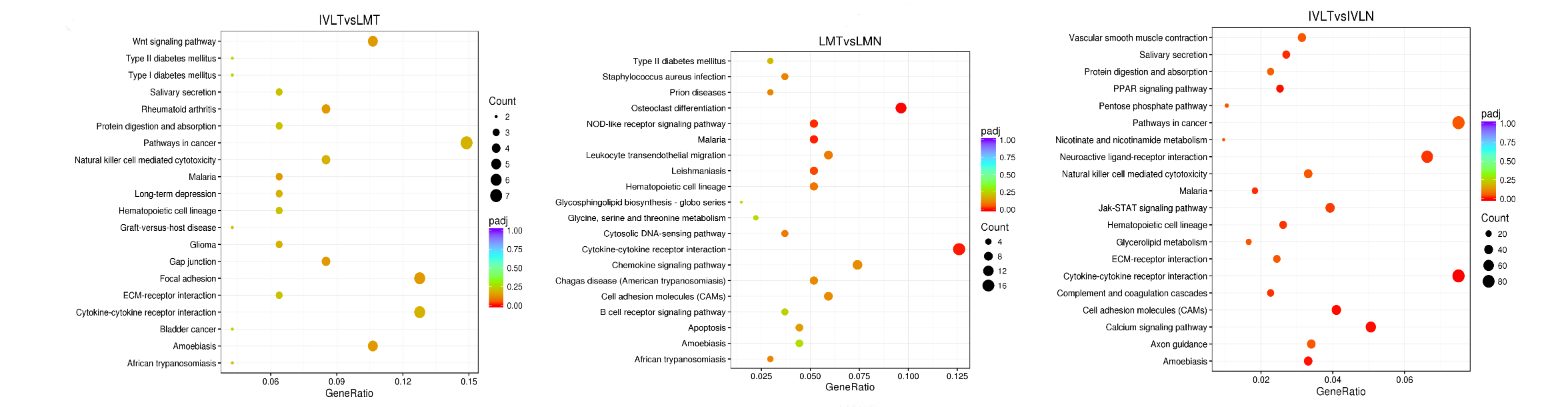


**Supplements** 10. **Plots for KEGG pathway analysis of term A, B and C (from left to right).**


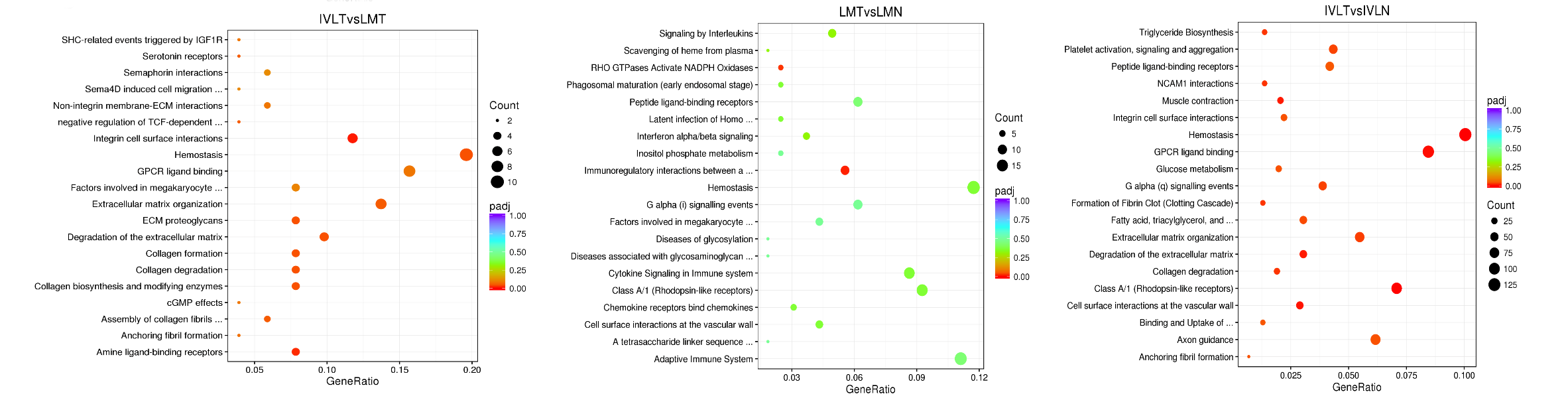


**Supplements** 11. **Plots for Reactome enrichment analysis of term A, B and C (from left to right).**
